# Metagenomic insights into the characteristics of soil microbial communities in the decomposing biomass of Moso bamboo forests under different management practices

**DOI:** 10.3389/fmicb.2022.1051721

**Published:** 2022-12-15

**Authors:** Xiaoping Zhang, Zhiyuan Huang, Zheke Zhong, Qiaoling Li, Fangyuan Bian, Chuanbao Yang

**Affiliations:** ^1^Key Laboratory of Bamboo Forest Ecology and Resource Utilization of National Forestry and Grassland Administration, China National Bamboo Research Center, Zhejiang, China; ^2^National Long-term Observation and Research Station for Forest Ecosystem in Hangzhou-Jiaxing-Huzhou Plain, Zhejiang, China; ^3^Engineering Research Center of Biochar of Zhejiang Province, Zhejiang, China

**Keywords:** CAZyme, plant-derived components, microbial-derived components, forest management, carbon sequestration

## Abstract

**Introduction:**

Considering the rapid growth and high biomass productivity, Moso bamboo (*Phyllostachys edulis*) has high carbon (C) sequestration potential, and different management practices can strongly modify its C pools. Soil microorganisms play an important role in C turnover through dead plant and microbial biomass degradation. To date, little is known about how different management practices affect microbial carbohydrate-active enzymes (CAZymes) and their responses to dead biomass degradation.

**Methods:**

Based on metagenomics analysis, this study analyzed CAZymes in three comparable stands from each Moso bamboo plantation: undisturbed (M0), extensively managed (M1), and intensively managed (M2).

**Results:**

The results showed that the number of CAZymes encoding plant-derived component degradation was higher than that encoding microbe-derived component degradation. Compared with the M0, the CAZyme families encoding plant-derived cellulose were significantly (*p* < 0.05) high in M2 and significantly (*p* < 0.05) low in M1. For microbe-derived components, the abundance of CAZymes involved in the bacterial-derived peptidoglycan was higher than that in fungal-derived components (chitin and glucans). Furthermore, M2 significantly increased the fungal-derived chitin and bacterial-derived peptidoglycan compared to M0, whereas M1 significantly decreased the fungal-derived glucans and significantly increased the bacterial-derived peptidoglycan. Four bacterial phyla (Acidobacteria, Actinobacteria, Proteobacteria, and Chloroflexi) mainly contributed to the degradation of C sources from the plant and microbial biomass. Redundancy analysis (RDA) and mantel test suggested the abundance of CAZyme encoding genes for plant and microbial biomass degradation are significantly correlated with soil pH, total P, and available K. Least Squares Path Modeling (PLS-PM) showed that management practices indirectly affect the CAZyme encoding genes associated with plant and microbial biomass degradation by regulating the soil pH and nutrients (total N and P), respectively.

**Discussion:**

Our study established that M2 and M1 impact dead biomass decomposition and C turnover, contributing to decreased C accumulation and establishing that the bacterial community plays the main role in C turnover in bamboo plantations.

## Introduction

Forest ecosystems are the most important carbon (C) pools and sinks in terrestrial ecosystems (Ameray et al., 2021), and store approximately two-thirds of the soil organic C (SOC) in terrestrial ecosystems (Canadell Josep et al., 2007). The forest soil C pool mainly comprises C allocated to the soil by tree roots and C contained in dead plant biomass (López-Mondéjar et al., 2020; Feng et al., 2022). Plant residues mainly consist of cellulose, hemicelluloses, and lignin, which form a complex and recalcitrant matrix (Mansora et al., 2019). Microbial biomass (e.g., bacterial and fungal cell walls) represents another important organic C pool (Wang et al., 2021; Patoine et al., 2022). The biomass of fungal residues mainly contains polysaccharides, which account for 80–90% of the total cell wall (Baldrian et al., 2013; Gow and Lenardon, 2022). Although bacterial cell wall composition can vary substantially (Silhavy et al., 2010), peptidoglycan is typically the main component (Egan et al., 2017; Apostolos and Pires, 2022).

Soil microorganisms are an important link between soil C input and output (Liang et al., 2017; Bhattacharyya et al., 2022), playing a critical role in C balance through dead biomass decomposition (López-Mondéjar et al., 2018). Žifčáková et al. (2017) suggested that the dead biomass turnover can be traced by studying the microbial carbohydrate-active enzymes (CAZymes) that mediate their C cycle. Among the CAZymes, glycoside hydrolases (GHs) and auxiliary activities (AAs) are associated with the decomposition of polysaccharides and lignin, respectively (López-Mondéjar et al., 2018; Lladó et al., 2019). Cellulases, β-glucosidases, and hemicellulases from GH families have been reported as the main enzymes degrading plant biomass (Bomble et al., 2017), whereas lysozymes and chitinases from GH families are linked to dead biomass degradation from microbial communities (Žifčáková et al., 2017). Ren et al. (2021) showed that the bacterial phyla Actinobacteria, Proteobacteria, and Acidobacteria dominated plant and microbial dead biomass decomposition through their corresponding CAZyme families. Members of Chloroflexi taxa are related to the degradation of plant compounds, such as cellulose, starch, and long-chain sugars (Li et al., 2020). Although CAZymes have been previously studied in forest soils, the mechanisms of C degradation driven by microbial communities are yet to be elucidated.

Moso bamboo (*Phyllostachys edulis*) is a typical representative forest resource in China, occupying 4.68 million hectares and accounting for approximately 72.96% of all the bamboo forests in the forested areas of China (Li and Feng, 2019). This bamboo species is an important non-wood forest product in China (Fei, 2021) due to its rapid growth, strong regeneration ability (Song et al., 2016), and C sequestration potential (Yen and Lee, 2011). A previous study confirmed that Moso bamboo forests are characterized by a higher C sequestration rate (8.13 Mg ha^−1^ year^−1^) than Chinese fir forests (3.35 Mg ha^−1^ year^−1^). Two management practices are used in Moso bamboo plantations: intensive management (such as fertilizer application, tillage, and removal of understory vegetation) and extensive management (including selective and regular harvesting of bamboo stems and shoots) (Yang et al., 2019). Previous studies have also found a relationship between different management approaches and soil C dynamics in Moso bamboo plantations (Li et al., 2013; Yang et al., 2019; Zhang et al., 2022). Specifically, Li et al. (2013) indicated that long-term intensive management practice decreases SOC storage and alters SOC chemical compositions, such as increased alkyl C and carbonyl C contents and decreased O-alkyl C and aromatic C contents. In contrast, extensive management can promote the accumulation of recalcitrant organic materials while decreasing C mineralization rates (Yang et al., 2019). However, the characteristics of the CAZymes in bamboo forests remain unclear.

This study describes an enzymatic toolbox that aids in the microbial decomposition of various biomass types. Soil samples from three different management practices were collected, and metagenomics was used to analyze the enzymatic tools of microbial decomposers. Moreover, we hypothesized that there were categories of CAZyme families involved in plant- and microbial-derived biomass decomposition and that the bacterial community contributes more to the dead biomass degradation in the Moso bamboo plantations. This study aimed to: (i) elucidate the distribution of the microbial CAZyme pool, (ii) characterize microbial taxa contribution to CAZyme genes related to the degradation of plant- and microbe-derived components, and (iii) examine the relationships between environmental parameters and specific CAZyme families.

## Materials and methods

### Experimental site and sample collection

The soil samples and their physiochemical properties used in this study were recently published (Zhang et al., 2022). The study area was Anji County (30°31′–30°14′N, 119°37–119°15′ E) in Zhejiang Province, China. A previous study presented meteorological data for the study area (Yang et al., 2019). Moso bamboo plantations under three different management strategies were selected: unmanaged (M0), extensively managed (M1), and intensively managed (M2). The M0 bamboo plantation had not been managed for >60 years (recoded by the Anji County Forestry Bureau), and had gradually developed into a mixed forest; the M1 bamboo plantation had been subjected to some management practices including selective and regular harvest of bamboo trunks, and shoots every 2 years; the M2 practices included selective harvesting, understory vegetation removal, and annual fertilization (nitrogen [N], 300–500 kg·ha^−1^; phosphorus [P], 50–200 kg·ha^−1^; and potassium [K], 100–250 kg·ha^−1^) during mid-to-late June. More information regarding the Moso bamboo plantations can be found in a study by Yang et al. (2019).

Three sites in the study area (3 × 2 km) were chosen for investigation in May 2021 based on similarities to the initial site conditions found in previous studies (Yang et al., 2019; Zhang et al., 2022). At each site, three comparable stands (M0, M1, and M2) with similar forest-land characteristics, such as soil type, elevation, slope gradient, and other features, were chosen. Three 20 × 20 m plots were established in each selected bamboo stand for sampling. A composite sample within each plot was obtained from five different points at depths of 0–20 cm. Fresh soil samples were sieved (mesh size of 2 mm) to remove stones, roots, and large organic residues. Subsequently, they were divided into two parts: one part was stored at −80°C for metagenomics, and the other was air-dried to analyze physicochemical properties.

### Soil chemical analysis

A glass electrode was used to measure the soil pH at a soil/water ratio of 1:2.5. SOC was determined using a total organic C (TOC) analyzer (Multi N/C 3100; Analytik Jena, Germany). Soil total N (TN, the Kjeldahl method), total P (TP, ammonium molybdate method), and available K (AK, extracted using 1 mol·L^−1^ ammonium acetate acid); were determined according to Lu (2000). Detailed information on the soil factors at these sampling sites is shown in Supplementary Table S1.

### DNA extraction and sequencing

Soil DNA was extracted using the FastDNA SPIN kit (MP Biomedical, Santa Anna, CA, United States), according to the manufacturer’s instructions. The quality and concentration of the DNA extracts were assessed using a NanoDrop 2000. Library construction and Illumina NovaSeq 6,000 sequencing were conducted at the Shanghai Majorbio Bio-Pharm Technology Co., Ltd.

### Metagenome sequencing and analysis

Raw reads (about 12 Gb nucleotides for each sample) from the metagenome sequencing were processed to obtain quality-filtered reads for further analysis. The adaptor sequences and low-quality reads were removed using fastp version 0.20.0 (Chen et al., 2018) on the online platform of Majorbio Cloud Platform^1^ (Ren et al., 2022). Subsequently, contigs were assembled using the MEGAHIT assembler (Li et al., 2015) (parameters: kmer_min = 47, kmer_max = 97, step = 10), which uses succinct de Bruijn graphs. Contigs with a minimum size of 300 base pairs (bp) were selected for the final assembly. The open reading frames (ORFs) were identified using MetaGene (Noguchi et al., 2006), and the predicted ORFs over 100 bp in length were translated into amino acid sequences using the National Center for Biotechnology Information (NCBI) translation table. All predicted genes with an identity and coverage ≥0.9 were clustered using the CD-HIT program (Fu et al., 2012). SOAPaligner (Li et al., 2008) was utilized to map the reads after quality control to non-redundant gene sets with 95% identity, and to calculate gene abundance. Representative sequences of non-redundant gene catalogs were annotated based on the NCBI NR database using the Basic Local Alignment Search Tool for Proteins (BLASTP), as implemented in DIAMOND v0.9.19, with an e-value cutoff of 1e^−5^ using Diamond (Buchfink et al., 2015) for taxonomic annotations. Carbohydrate-active enzyme annotation was conducted using hmmscan^2^ against the CAZyme database^3^ with an e-value cut-off of 1e^−5^.

### Statistical analyses

The abundance values in metagenomes normalized by transcripts per kilobase per million mapped reads (TPM). Non-metric multidimensional scaling (NMDS), permutational multivariate analysis of variance (PERMANOVA) and redundancy analysis (RDA) were performed in the ‘Vegan’ (Dixon, 2003) package based on Bray-Curtis distance. The circos plots were generated using the ‘circlize’ package (Gu et al., 2014). The R package‘ggcor’was used for Spearman’s correlation test and the Mantel test (Huang et al., 2020). Least Squares Path Modeling (PLS-PM) were performed with the R package ‘plspm’ (Sanchez et al., 2017). Contribution analysis was conducted on the Majorbio Cloud Platform^4^ (Ren et al., 2022). The significance of differences among soil samples was tested by One-way ANOVA followed by least significant difference (LSD) *post hoc* test.

## Results

### Changes in glycoside hydrolases and auxiliary enzyme families

**Figure 1:**
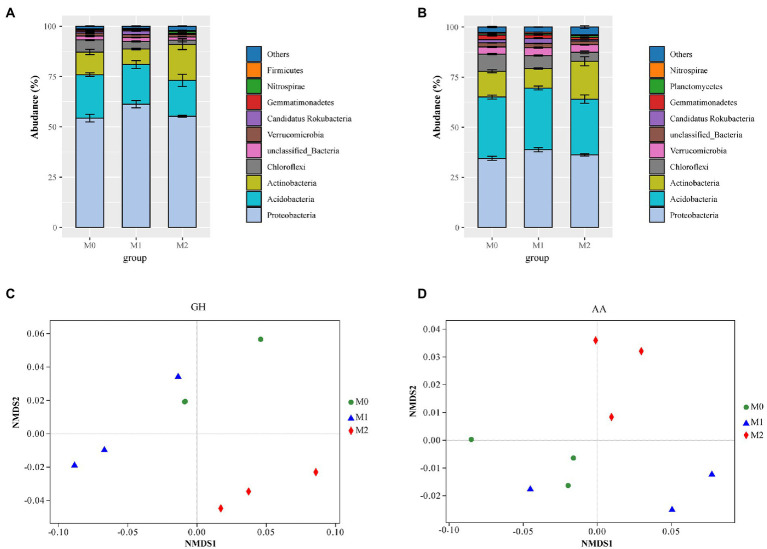
The abundance of microbial glycoside hydrolases (GHs) and auxiliary activities (AAs) in the bamboo plantations under different management practices. **(A,B)** contribution of microbial phyla to the GHs and AAs families; **(C,D)** Non-metric multidimensional scaling (NMDS) of the GH and AA families. M0, M1, and M2 indicates undisturbed, extensively managed, and intensively managed bamboo plantations, respectively.

In total, 7,759,044 CAZymes were identified from 13,509,440 predicted proteins in the metagenome. Of these, > 98% was assigned to bacteria (Supplementary Table S2). Moreover, of the total CAZyme pools, GHs and AAs represented average values of 29.38 and 10.69%, respectively (Supplementary Table S3). Most GHs were mainly attributed to Proteobacteria (56.95%), Acidobacteria (19.72%), Actinobacteria (12.30%), Chloroflexi (3.90%), and Verrucomicrobia (1.42%; Figure 1A), whereas AAs were largely attributed to Proteobacteria (36.48%), Acidobacteria (29.75%), Actinobacteria (13.84%), Chloroflexi (6.50%), Verrucomicrobia (3.71%), Candidatus Rokubacteria (1.69%), and Gemmatimonadetes (1.55%; Figure 1B). Compared to M0 and M1, M2 significantly increased GHs abundance, whereas there was no significant difference between M1 and M0 (Supplementary Table S3). In addition, no differences in AAs among the three groups were identified (Supplementary Table S3). The NMDS further revealed significant differences in GHs (PERMANOVA: R^2^ = 0.859, *p* = 0.005) and AA (PERMANOVA: R^2^ = 0.826, *p* = 0.01) families among the different management approaches (Figures 1C,D).

### Changes in specific CAZyme families involved in the degradation of plant, fungal, and bacterial biomass

**Figure 2:**
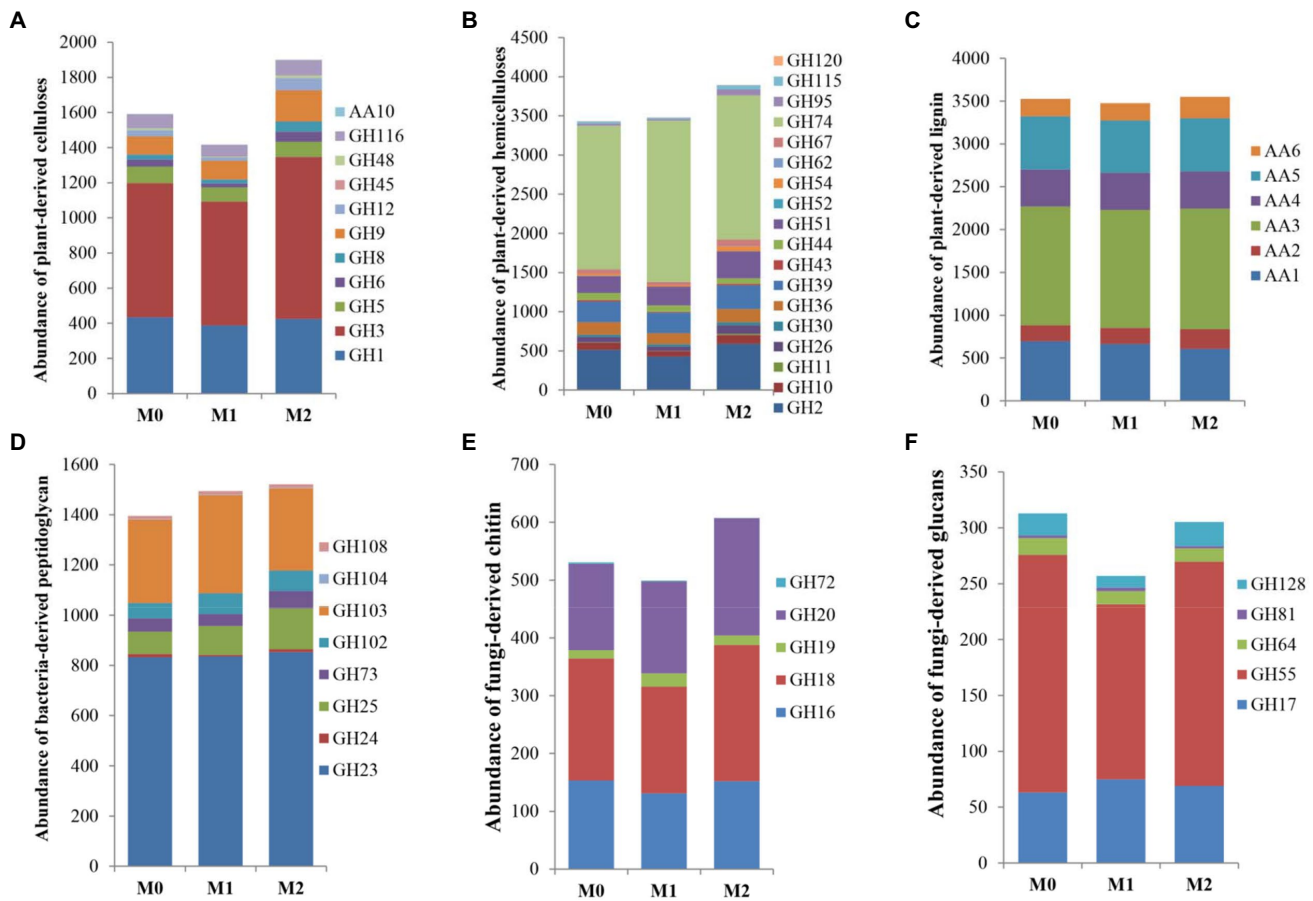
The abundance of selected GHs and AAs related to the degradation of the plant- and microbial- derived biomass in the bamboo plantations under different management practices. **(A–C)** plant-derived biomass decomposotion; **(D)** bacteria-derived biomass decomposition; **(E,F)** fungi-derived biomass decomposition.

The genes were assigned to the enzymatic activities involved in the degradation of plant- and microbial- compounds according to Ren et al. (2021). For plant biomass (Figure 2; Supplementary Table S4), M2 significantly increased the abundance of CAZyme families involved in plant-derived cellulose than M0 (*p* < 0.05), whereas it significantly decreased in M1 (*p* < 0.05); Among the three groups, no significant differences (*p* > 0.05) were observed in the CAZyme families involved in plant-derived hemicellulose and lignin. Additionally, M2 exhibited a significantly (*p* < 0.05) higher abundance of CAZyme families involved in fungi-derived chitin within fungal biomass than M0 and M1 (Figure 2; Supplementary Table S4). No significant difference (*p* > 0.05) was observed between M0 and M1. Moreover, M1 exhibited a significantly (*p* < 0.05) lower abundance of CAZyme families involved in fungi-derived glucans than M0 and M2 (Figure 2; Supplementary Table S4). There was no significant difference (p > 0.05) between M0 and M2 (Figure 2; Supplementary Table S4). Furthermore, M2 and M1 significantly increased (p < 0.05) the abundance of CAZyme families involved in bacteria-derived peptidoglycan than M0 (Figure 2; Supplementary Table S4).

**Figure 3:**
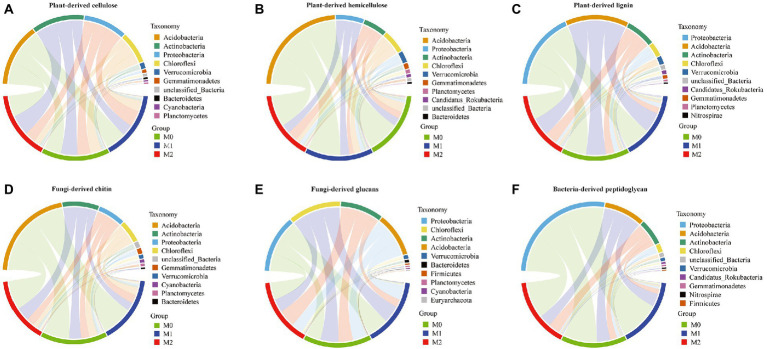
Contribution of the microbial phyla to microbial CAZyme genes for the dead biomass decomposing in the bamboo plantations. **(A–C)** plant-derived biomass decomposotion; **(D,E)** fungi-derived biomass decomposition; **(E,F)** bacteria-derived biomass decomposition.

The CAZyme families associated with the degradation of plant- and microbe-derived components were mainly assigned to four bacterial phyla: Acidobacteria, Actinobacteria, Proteobacteria, and Chloroflexi (Figure 3). In particular, Actinobacteria, Chloroflexi, and Proteobacteria exhibited significant differences among the three groups (Supplementary Table S5). The analysis of microbial phyla contribution to plant-derived biomass decomposition revealed that Actinobacteria was significantly increased in M2 than in M0 and significantly decreased in M1 (Supplementary Table S5). Chloroflexi exhibited a lower number of transcripts per million (TPM) in M1 and M2 than in M0, whereas Proteobacteria exhibited a significant difference among the three groups (plant-derived cellulose, M1 > M2 ≈ M0; plant-derived hemicellulose, M2 > M0; plant-derived lignin, M2 ≈ M1 > M0) (Supplementary Table S5). The three phyla were identified according to the contribution of microbial phyla to fungi-derived biomass decomposition in the following order: fungi-derived chitin (Actinobacteria, M2 > M0 > M1; Chloroflexi, M0 ≈ M1 > M2; and Proteobacteria, M2 > M0 ≈ M1) and fungi-derived glucans (Actinobacteria, M2 ≈ M0 > M1; Chloroflexi, M0 > M1 > M2; and Proteobacteria, M2 ≈ M1 ≈ M0) (Supplementary Table S5). Regarding the contribution of microbial phyla to bacteria-derived peptidoglycan decomposition, Actinobacteria was significantly higher in M2 than in M0 and M1, and Chloroflexi (M0 > M1 > M2) and Proteobacteria (M1 > M2 > M0) exhibited significant differences among the three groups (Supplementary Table S5).

Management practices altered specific families related to the degradation of plant- and microbe-derived components (Supplementary Table S6). For the plant-derived components, GH3 (β-glucosidase, 797.31 TMP, M2 > M0 > M1), GH74 (xyloglucanase, 1910.99 TMP, M1 > M2 ≈ M0), and AA3 (oxidase, 1390.84 TMP, M2 ≈ M1 ≈ M0) were the most abundant families involved in plant-derived cellulose, hemicellulose, and lignin decomposition, respectively (Supplementary Table S6). For the microbe-derived components, GH18 (chitinase, 210.26 TMP, M2 > M1), GH55 (exo-β-1, 3-glucanase/endo-1, 3-β-glucanase, 190.05 TMP, M0 ≈ M2 > M1), and GH23 (lysozyme, 840.24 TMP, M0 ≈ M1 ≈ M2) were the most abundant families involved in fungi-derived chitin and glucans, and bacteria-derived peptidoglycan decomposition, respectively (Supplementary Table S6).

### Relationship between the CAZymes involved in the degradation of dead biomass and soil properties

**Figure 4:**
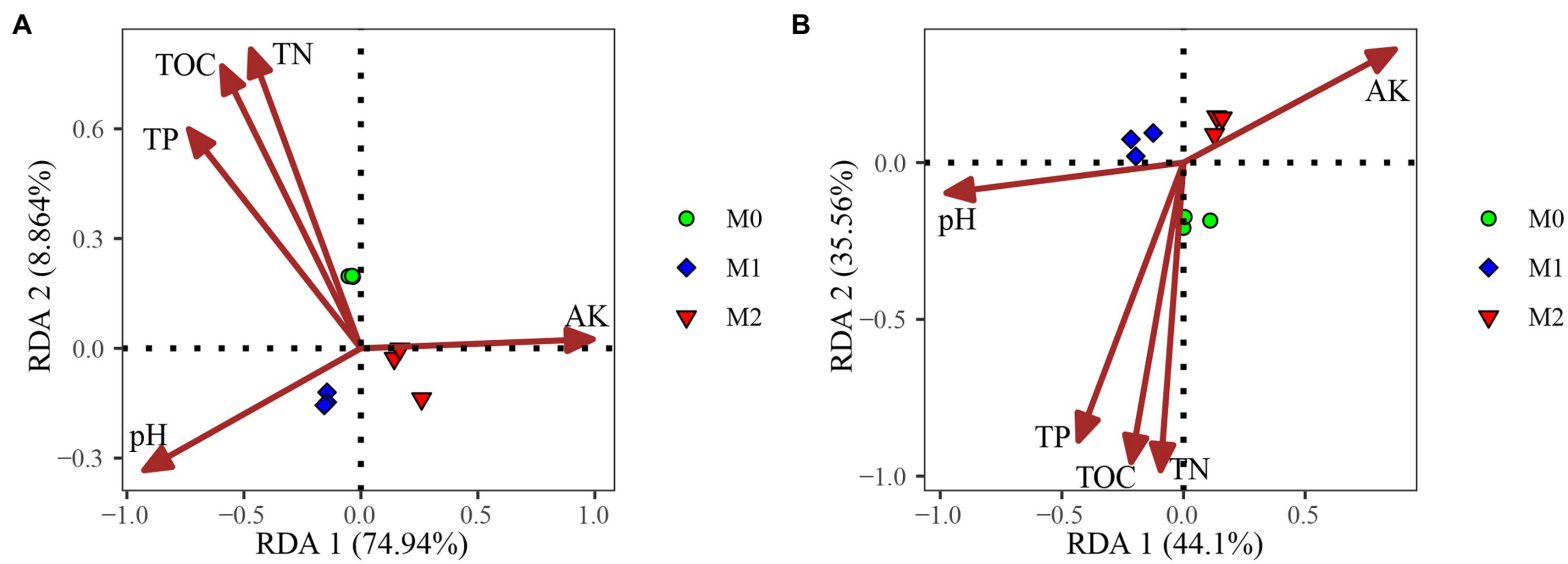
Redundancy analysis of soil properties and microbial CAZyme genes for the plant- **(A)** and microbial-derived **(B)** biomass decomposing in the bamboo plantations under different management practices.

RDA showed that the first two RDA axes accounted for 83.70 and 79.66%, respectively, of the total variation of CAZyme families related to plant and microbial biomass degradation (Figure 4). Soil pH, TOC, TN, TP, AK significantly affected the CAZyme families associated with plant and microbial biomass degradation (Figure 4). Furthermore, the Mantel test revealed that soil pH, TP, and AK were significantly correlated with the CAZyme families involved in the decomposition of plant and microbial biomass; soil TOC and TN were also significantly correlated with CAZyme families involved in the decomposition of microbial biomass (Table 1).

**Table 1:** Spearman’s correlation analysis and Mantel tests for microbial CAZyme families involved in the decomposition of the plant- and microbial-derived components against soil properties (9,999 permutations).

	Plant-derived components		Microbial-derived components	
	*r*	*p*-value	*r*	*p*-value
pH	0.552	0.015	0.78	0.0002
TOC	0.281	0.0583	0.366	0.0329
TN	0.237	0.0843	0.331	0.0384
TP	0.654	0.0081	0.477	0.0188
AK	0.876	0.0002	0.748	0.0009

**Figure 5:**
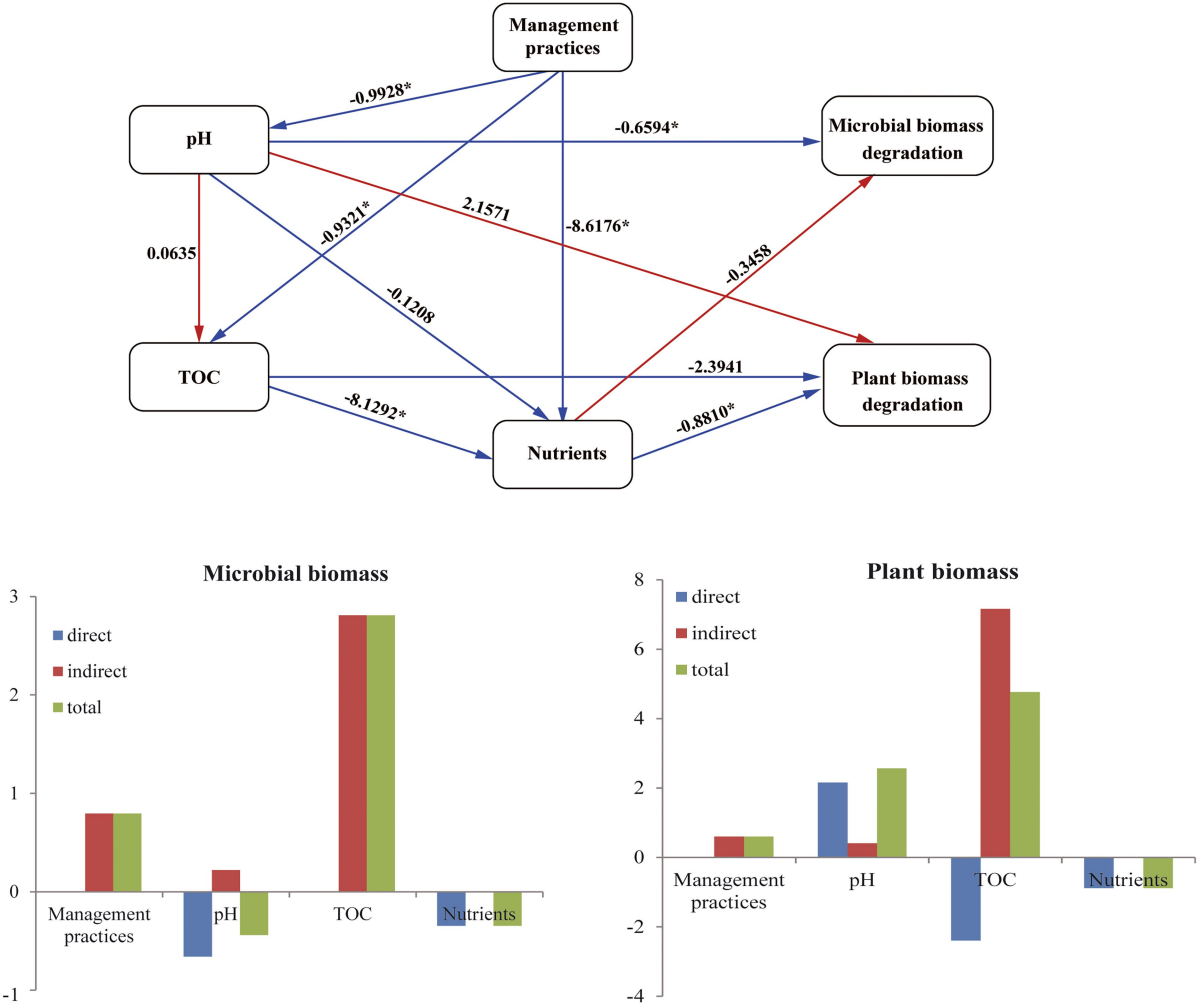
PLS-PM shows the effects of management practices on the microbial CAZyme genes for the plant- and microbial-derived biomass decomposing.

PLS-PM was implemented to assess the direct and indirect effects of management practices and soil properties on the CAZyme families (Figure 5). PLS-PM with a Goodness-of-Fit (GoF) index of 0.833 explained 82.2 and 73.5% of the variation for CAZyme families in plant and microbial biomass decomposition, respectively. The PLS-PM analysis indicated that the management practices had an indirect positive effect on the plant and microbial biomass degradation by changing soil nutrients (TN and TP) (path coefficient = −0.881) and pH (path coefficient = −0.993), respectively. Management practices also indirectly affected the soil nutrients (TN and TP) *via* altering soil TOC (path coefficient = −8.129).

## Discussion

### Effects of management practices on microbial CAZyme genes in the bamboo plantations

Our findings suggest that soil microbes contain numerous CAZymes which are involved in plant and microbial biomass degradation, and facilitates C utilization in bamboo plantations. This result supports previous findings on soil microbes as the primary consumers of simple and recalcitrant substrates (Kramer et al., 2016; López-Mondéjar et al., 2018). Furthermore, the number of CAZymes associated with plant-derived component degradation was greater than that associated with microbe-derived components (Figure 2). This indicates that dead plant biomass contributes more to the soil C pool in bamboo plantations. This finding is consistent with previous findings that plant biomass is rich in C and can enrich the soil through above- and underground litter, which is the primary source of soil organic matter (SOM) (Castellano et al., 2015). Our findings also showed that the forest management practices influenced soil microbial CAZyme families involved in plant-derived cellulose but not plant-derived hemicellulose and lignin. These results suggest that plant-derived cellulose is a key factor determining SOC accumulation in bamboo plantations. Moreover, microbial CAZyme families involved in plant-derived cellulose were significantly increased in M2 than in M0 but decreased in M1, indicating that more disturbance promotes the degradation of plant-derived cellulose. A significant correlation was discovered between soil pH and AK in CAZyme families associated with plant-derived cellulose degradation. Therefore, the soil pH and AK variations partially contributed to the changes in the CAZyme families associated with plant-derived cellulose degradation.

Our results revealed that the abundance of genes involved in the decomposition of bacteria-derived biomass was greater than that of fungi-derived biomass. This indicates that bacteria-derived biomass degradation for C cycling is more important than fungi-derived biomass. Our findings are consistent with Gunina et al. (2017), who reported that dead bacterial biomass had a higher turnover rate than fungal biomass. Egan et al. (2017) also demonstrated that bacterial-derived peptidoglycan is the main and universal component of the cell wall that changes rapidly. This study found that M2 and M1 significantly increased the abundance of soil microbial CAZyme families involved in bacterial-derived peptidoglycan, indicating that forest management can increase the decomposition rate of bacterial-derived peptidoglycan. Ren et al. (2021) suggested that the soil environment and substrates influence the decomposition of microbe-derived components in forest soils. In this study, CAZyme families involved in bacteria-derived peptidoglycan degradation were negatively correlated with soil TOC, TN, and TP. Thus, the changes in soil properties (especially TOC, TN, and TP) contributed to the variations in the CAZyme families involved in bacteria-derived peptidoglycan degradation.

M1 exhibited a lower abundance in the CAZyme families involved in fungi-derived biomass than M2. According to Clemmensen et al. (2013), some fungal biomass fractions are highly recalcitrant and are likely a major source of recalcitrant SOM. Combined with the results for the CAZyme families involved in plant-derived cellulose, M1 can alleviate the degradation of SOC relative to M2. Similar to the results of Yang et al. (2019), who found that the rate of C mineralization was highest in M2 and lowest in M1, indicating M0 and M1 had much higher potential in terms of soil C sequestration than M2.

### Microbial CAZymes for metabolic activity in bamboo plantations under different management practices

This study found that bacterial communities accounted for >98% of the microbial community in the bamboo soils studied. This result suggests that the bacterial community plays important roles in the degradation of dead plants and microbial biomass, partly because bacteria produce enzymes and participate in the degradation of cellulose, hemicellulose, and chitin (Eichorst and Kuske, 2012; López-Mondéjar et al., 2016). The microbial CAZyme families associated with the degradation of dead plants and microbial biomass were mainly assigned to four bacterial phyla: Acidobacteria, Actinobacteria, Proteobacteria, and Chloroflexi. Members of the Actinobacteria produce extracellular enzymes and play a role in the degradation of plant polysaccharides and phenolic compounds (Mccarthy, 1987; Warren, 1996). Notably, the analysis of the contribution of microbial phyla to plant-derived cellulose degradation revealed that these four phyla were significantly altered. Actinobacteria and some Acidobacteria are oligotrophic bacteria (Kielak et al., 2016; Liu et al., 2018), affecting the decomposition of organic matter under limited nutrient conditions (Fierer et al., 2007; Banerjee et al., 2016). Therefore, the increased Actinobacteria and Acidobacteria indicated low nutrient levels in M2. Members of Proteobacteria were related to N fixation, organic matter degradation, and plant growth improvement (Yarwood et al., 2009; Delmont et al., 2018). Several studies reported that Proteobacteria are rich in higher pH soils (Christian et al., 2009; Chu et al., 2010). The increased pH can clarify the higher abundance of Proteobacteria in M1 than in M0 and M2. The phylum Chloroflexi plays a role in cycling C and N (Hug et al., 2013). Thus, the increased abundance of Chloroflexi was related to the higher TOC and TN contents in M0 than in M1 and M2.

### Factors driving the CAZyme families involved in plant and microbial biomass degradation

Several studies have found that soil properties are one of the most important factors influencing soil microbial diversity and function (Zhang et al., 2021; Wang et al., 2022). According to Cardenas et al. (2015), soil properties can exert selective pressure on soil microorganisms, shaping changes in CAZyme coding genes. In our study, RDA and mantel tests showed that soil pH, TP, and AK content significantly affected the abundance of CAZyme encoding genes. Results of PLS-PM suggested that management practices altered the CAZyme families during the decomposition of plant and microbial biomass by changing the soil nutrients (TN and TP) and pH, respectively. These results indicated soil pH and TP were the major factors shaping the specific CAZyme families. Studies have shown that pH is the vital factor shaping soil microbial communities (Qi et al., 2018; Lopes et al., 2021). Conversely, soil pH indirectly affects microbial communities due to its close relationship with other soil factors, such as fertility and nutrient availability (Christian et al., 2009; Penn and Camberato, 2019). Phosphorus is an important macronutrient for all biota on Earth, and changes therein affect the microbial community (Bergkemper et al., 2016; Samaddar et al., 2019).

## Conclusion

This study elucidated the trends of microbial CAZymes in Moso bamboo stands. The abundance of CAZymes targeting dead plant biomass was higher than that of dead microbial biomass, indicating that dead plant biomass was the major source of the soil C pool in the bamboo plantations. Management practices alter the abundance of microbial CAZymes encoding plant- and microbial-derived biomass degradation, further affecting the C accumulation. The dominant microorganisms for microbial C degradation were bacterial communities, suggesting that the bacterial community contributes more to the degradation of microbial C in the bamboo soil and that Acidobacteria, Actinobacteria, Proteobacteria, and Chloroflexi, in particular, are essential for microbial C decomposition. Overall, our findings expand the understanding of the relationship between microbial CAZyme families and C decomposition, establishing the importance of the bacterial community for C cycling in bamboo plantations.

## Data availability statement

The data presented in the study are deposited in the National Center for Biotechnology Information (NCBI) BioProject repository, accession number PRJNA883525. https://www.ncbi.nlm.nih.gov/bioproject/PRJNA883525.

## Author contributions

XZ and ZZ: conceptualization and methodology. XZ, ZH, QL, FB, and CY: investigation. XZ: writing—original draft preparation. ZZ: writing—review and editing. XZ and ZZ: funding acquisition. All authors contributed to the article and approved the submitted version.

## Funding

This research was supported by the People’s Government of Zhejiang Province−Chinese Academy of Forestry cooperative project (2020SY01), and the Fundamental Research Funds of CAF (CAFYBB2021QB007).

## Conflict of interest

The authors declare that the research was conducted in the absence of any commercial or financial relationships that could be construed as a potential conflict of interest.

## Publisher’s note

All claims expressed in this article are solely those of the authors and do not necessarily represent those of their affiliated organizations, or those of the publisher, the editors and the reviewers. Any product that may be evaluated in this article, or claim that may be made by its manufacturer, is not guaranteed or endorsed by the publisher.

## Footnotes

^1^ https://cloud.majorbio.com/

^2^ http://hmmer.janelia.org/search/hmmscan

^3^ http://www.cazy.org/

^4^ http://www.majorbio.com
