# Supplementary material for: Metagenomic insights into the characteristics of soil microbial communities in the decomposing biomass of Moso bamboo forests under different management practices
Source: Front Microbiol. 2022 Dec 15;13:1051721. doi: 10.3389/fmicb.2022.1051721 (PMC9797724; doi:10.3389/fmicb.2022.1051721)
Supplement: Supplementary file 1 [file Table_1.DOCX]

**Table S1.** Soil properties in bamboo soils under different management practices

|  | M0 | M1 | M2 |
| --- | --- | --- | --- |
| pH | 4.92 ± 0.02 b | 5.24 ± 0.04 a | 4.69 ± 0.01 c |
| TOC (g/kg) | 79.87 ± 2.33 a | 47.06 ± 0.92 b | 22.67 ± 0.08 c |
| TN (g/kg) | 6.56 ± 0.59 a | 3.63 ± 0.10 b | 2.25 ± 0.44 c |
| TP (g/kg) | 1.27 ± 0.01 a | 1.17 ± 0.01 b | 0.99 ± 0.01 c |
| AK (mg/kg) | 19.60 ± 1.46 b | 14.35 ± 0.52 c | 30.39 ± 1.46 a |

TOC, total organic carbon; TN, total nitrogen; TP, total Phosphorus; AK, available K; M0, M1, and M2, undisturbed, extensively managed, and intensively managed bamboo stands. Different lowercase letters within rows indicate significant difference at p < 0.05.

**Table S2.** The abundance of microbial community at the kingdom level

|  | Bacteria | Archaea | Eukaryota | unclassified | Total |
| --- | --- | --- | --- | --- | --- |
| M0-1 | 231068 | 3862 | 46 | 60 | 235036 |
| M0-2 | 262222 | 5036 | 72 | 74 | 267404 |
| M0-3 | 231254 | 4060 | 56 | 94 | 235464 |
| M1-1 | 254152 | 3156 | 60 | 138 | 257506 |
| M1-2 | 202662 | 2452 | 92 | 54 | 205260 |
| M1-3 | 213968 | 2882 | 66 | 66 | 216982 |
| M2-1 | 248012 | 3242 | 52 | 102 | 251408 |
| M2-2 | 235202 | 1992 | 44 | 78 | 237316 |
| M2-3 | 247554 | 2336 | 62 | 56 | 250008 |

**Table S3.** The abundance of genes (TPM: transcripts per kilobase per million mapped reads) in the whole metagenome encoding CAZymes.

|  | AA | CBM | CE | GH | GT | PL | Total |
| --- | --- | --- | --- | --- | --- | --- | --- |
| M0-1 | 98022 | 17724 | 156664 | 259974 | 322650 | 17132 | 872166 |
| M0-2 | 111806 | 19906 | 176354 | 299620 | 374100 | 20294 | 1002080 |
| M0-3 | 97362 | 17034 | 151788 | 256996 | 322048 | 16374 | 861602 |
| M1-1 | 103148 | 17258 | 161510 | 266362 | 343854 | 19044 | 911176 |
| M1-2 | 80598 | 13846 | 130980 | 213676 | 273316 | 15480 | 727896 |
| M1-3 | 85124 | 14848 | 137706 | 228946 | 293182 | 17216 | 777022 |
| M2-1 | 92836 | 19918 | 156928 | 297720 | 334604 | 19510 | 921516 |
| M2-2 | 88498 | 16314 | 143742 | 254210 | 296146 | 14524 | 813434 |
| M2-3 | 94490 | 17496 | 151776 | 273946 | 318908 | 15536 | 872152 |

GH: Glycoside Hydrolases, AA: Auxiliary Activities, CBM: Carbohydrate-Binding Modules, CE: Carbohydrate Esterases, PL: Polysaccharide Lyases, GT: GlycosylTransferases.

**Table S4.** Comparison and analysis of the abundance of selected GHs and AAs involved in the degradation of the dead biomass

|  |  | M0 | M1 | M2 |
| --- | --- | --- | --- | --- |
| Plant  biomass | Cellulose | 1591.33 ± 27.41 b | 1416.73 ± 33.01 c | 1900.36 ± 57.07 a |
|  | Hemicellulose | 3432.57 ± 41.21 a | 3481.61 ± 55.30 a | 3896.29 ± 478.49 a |
|  | Lignin | 3525.15 ± 25.80 a | 3475.22 ± 45.32 a | 3549.17 ± 66.74 a |
| Fungi  biomass | Chitin | 530.51 ± 5.97 b | 499.13 ± 36.15 b | 607.65 ± 50.12 a |
|  | Glucans | 312.91 ± 16.12 a | 257.03 ± 5.46 b | 305.36 ± 28.98 a |
| Bacteria  biomass | Peptidoglycan | 1395.27 ± 23.47 b | 1493.75 ± 8.47 a | 1520.60 ± 34.15 a |

M0, M1, and M2, undisturbed, extensively managed, and intensively managed bamboo stands. Different lowercase letters within rows indicate significant difference at p < 0.05.

**Table S5.** Abundance of microbial phyla encoding the decomposition of the plant- and microbial-derived components in the bamboo plantations under different management practices. Different letters indicate significant differences.

| Group | Taxa | M0 | M1 | M2 |
| --- | --- | --- | --- | --- |
| plant-derived cellulose | Acidobacteria | 221.22 ± 13.61 b | 226.21 ± 7.34 b | 321.68 ± 53.37 a |
|  | Actinobacteria | 214.45 ± 4.76 b | 126.60 ± 9.41 c | 312.56 ± 36.63 a |
|  | Proteobacteria | 167.49 ± 2.18 b | 180.06 ± 6.38 a | 168.26 ± 5.36 b |
|  | Chloroflexi | 157.12 ± 3.44 a | 119.89 ± 3.11 b | 99.60 ± 22.14 b |
|  | Verrucomicrobia | 23.75 ± 0.52a | 19.32 ± 0.97a | 28.81 ± 8.31a |
|  | Gemmatimonadetes | 12.89 ± 1.66 a | 13.70 ± 0.16 a | 8.34 ± 2.99 b |
|  | unclassified_Bacteria | 7.07 ± 0.34 c | 11.53 ± 0.17 a | 8.93 ± 0.91 b |
|  | Bacteroidetes | 7.43 ± 1.55a | 6.41 ± 0.67a | 8.58 ± 3.73a |
|  | Cyanobacteria | 6.29 ± 1.56 a | 3.13 ± 0.72 b | 4.48 ± 0.97 ab |
|  | Planctomycetes | 3.99 ± 1.47 b | 3.00 ± 1.40 b | 6.90 ± 1.11 a |
|  | others | 19.05 ± 1.63 b | 24.98 ± 0.77 a | 24.31 ± 3.68 a |
| plant-derived hemicellulose | Acidobacteria | 864.93 ± 29.03a | 895.27 ± 34.72a | 949.49 ± 187.31a |
|  | Proteobacteria | 216.59 ± 12.87 b | 245.77 ± 20.43 ab | 269.93 ± 18.65 a |
|  | Actinobacteria | 189.45 ± 6.55 b | 123.15 ± 5.27 c | 307.89 ± 19.81 a |
|  | Chloroflexi | 258.71 ± 9.58 a | 200.63 ± 9.46 b | 127.60 ± 23.19 c |
|  | Verrucomicrobia | 65.33 ± 5.67 b | 100.10 ± 9.27 ab | 127.16 ± 33.64 a |
|  | Gemmatimonadetes | 45.39 ± 5.08a | 57.67 ± 6.75a | 36.28 ± 14.57a |
|  | Planctomycetes | 24.31 ± 2.16 b | 27.54 ± 2.58 b | 40.32 ± 4.18 a |
|  | Candidatus_Rokubacteria | 18.86 ± 1.46 b | 39.97 ± 2.42 a | 10.57 ± 2.31 c |
|  | unclassified_Bacteria | 23.10 ± 0.64 b | 19.44 ± 2.16 c | 26.82 ± 1.93 a |
|  | Bacteroidetes | 12.87 ± 2.57a | 11.66 ± 1.05a | 21.22 ± 15.30a |
|  | others | 74.47 ± 7.12 b | 64.66 ± 1.60 b | 92.75 ± 8.09 a |
| plant-derived lignin | Proteobacteria | 646.11 ± 13.25 b | 702.79 ± 23.73 a | 678.97 ± 3.42 a |
|  | Acidobacteria | 574.83 ± 20.28a | 558.41 ± 16.94a | 521.29 ± 43.72a |
|  | Actinobacteria | 241.14 ± 13.30 b | 176.89 ± 4.98 c | 354.24 ± 42.24 a |
|  | Chloroflexi | 160.23 ± 6.02 a | 117.33 ± 4.51 b | 83.90 ± 12.82 c |
|  | Verrucomicrobia | 66.68 ± 6.44a | 70.30 ± 5.56a | 69.49 ± 4.89a |
|  | unclassified_Bacteria | 39.09 ± 1.89a | 37.98 ± 3.58a | 35.29 ± 1.99a |
|  | Candidatus_Rokubacteria | 30.43 ± 0.60 b | 47.67 ± 4.46 a | 15.18 ± 0.49 c |
|  | Gemmatimonadetes | 40.65 ± 6.83 a | 28.42 ± 2.22 b | 17.19 ± 4.82 c |
|  | Planctomycetes | 12.42 ± 1.23 b | 13.75 ± 0.97 b | 18.67 ± 2.57 a |
|  | Nitrospirae | 12.06 ± 1.64 a | 13.27 ± 1.40 a | 7.70 ± 0.72 b |
|  | others | 52.30 ± 6.43 b | 46.10 ± 1.99 b | 72.57 ± 12.86 a |
| fungi-derived chitin | Acidobacteria | 113.63 ± 6.91a | 122.65 ± 11.00a | 147.30 ± 25.48a |
|  | Actinobacteria | 52.20 ± 1.55 b | 32.41 ± 0.81 c | 67.60 ± 9.63 a |
|  | Proteobacteria | 35.33 ± 1.90 b | 34.55 ± 2.65 b | 41.27 ± 1.83 a |
|  | Chloroflexi | 35.92 ± 0.63 a | 30.47 ± 2.42 a | 22.49 ± 4.38 b |
|  | unclassified_Bacteria | 7.87 ± 0.54a | 7.13 ± 1.48a | 9.28 ± 1.65a |
|  | Gemmatimonadetes | 9.00 ± 1.78a | 8.29 ± 1.16a | 4.51 ± 3.15a |
|  | Verrucomicrobia | 3.79 ± 0.54a | 5.01 ± 2.24a | 6.20 ± 4.29a |
|  | Cyanobacteria | 6.68 ± 0.47 a | 2.30 ± 0.64 b | 3.30 ± 0.66 b |
|  | Planctomycetes | 1.99 ± 0.15 b | 3.43 ± 0.63 ab | 4.96 ± 1.18 a |
|  | Bacteroidetes | 2.18 ± 0.52a | 1.79 ± 0.27a | 2.52 ± 0.58a |
|  | others | 7.66 ± 1.62a | 8.18 ± 2.43a | 5.13 ± 0.36a |
| fungi-derived glucans | Proteobacteria | 37.51 ± 4.20a | 42.35 ± 2.93a | 41.65 ± 5.26a |
|  | Chloroflexi | 46.02 ± 1.67 a | 35.31 ± 0.96 b | 32.04 ± 1.12 c |
|  | Actinobacteria | 37.39 ± 3.31 a | 15.73 ± 0.41 b | 41.12 ± 4.49 a |
|  | Acidobacteria | 36.04 ± 6.44a | 30.43 ± 2.87a | 27.64 ± 2.25a |
|  | Verrucomicrobia | 0.92 ± 0.30 b | 1.77 ± 0.85 b | 5.00 ± 2.32 a |
|  | Bacteroidetes | 1.74 ± 0.40a | 1.78 ± 0.78a | 1.81 ± 0.63a |
|  | Firmicutes | 1.06 ± 0.60a | 0.29 ± 0.28a | 1.01 ± 0.28a |
|  | Planctomycetes | 0.04 ± 0.06 b | 1.07 ± 0.24 a | 0.96 ± 0.17 a |
|  | Cyanobacteria | 1.24 ± 0.32 a | 0.32 ± 0.29 b | 0.11 ± 0.19 b |
|  | Euryarchaeota | 0.53 ± 0.46a | 0.00 ± 0.00a | 0.75 ± 0.49a |
|  | others | 0.50 ± 0.35 b | 0.63 ± 0.29 b | 2.15 ± 0.64 a |
| bacteria-derived peptidoglycan | Proteobacteria | 399.89 ± 18.91 c | 474.92 ± 15.62 a | 434.95 ± 9.15 b |
|  | Acidobacteria | 159.04 ± 7.87a | 152.68 ± 14.49a | 140.44 ± 25.46a |
|  | Actinobacteria | 82.89 ± 8.42 b | 59.78 ± 2.57 b | 141.11 ± 19.06 a |
|  | Chloroflexi | 43.67 ± 2.02 a | 29.18 ± 1.93 b | 15.77 ± 5.97 c |
|  | unclassified_Bacteria | 14.58 ± 2.93a | 14.57 ± 1.09a | 13.19 ± 1.91a |
|  | Verrucomicrobia | 10.88 ± 1.66a | 11.93 ± 0.66a | 9.70 ± 0.37a |
|  | Candidatus_Rokubacteria | 5.47 ± 0.22 b | 14.15 ± 1.98 a | 0.79 ± 0.25 c |
|  | Gemmatimonadetes | 4.23 ± 1.39a | 4.36 ± 0.44a | 3.94 ± 1.88 a |
|  | Nitrospirae | 1.60 ± 0.83 c | 3.85 ± 0.46 b | 6.61 ± 0.49 a |
|  | Firmicutes | 3.85 ± 0.13 a | 1.45 ± 0.58 b | 3.94 ± 0.80 a |
|  | others | 9.81 ± 1.56 b | 8.34 ± 1.80 b | 16.78 ± 1.45 a |

**Table S6.** Abundance of selected microbial CAZyme families involved in the decomposition of the plant- and microbial-derived components in the bamboo plantations under different management practices. Different letters indicate significant differences.

| Group | Taxa | M0 | M1 | M2 |
| --- | --- | --- | --- | --- |
| plant-derived cellulose | GH1 | 433.87 ± 0.45 a | 387.55 ± 16.46 b | 424.98 ± 4.79 a |
|  | GH3 | 763.94 ± 26.26 b | 705.07 ± 27.15 c | 922.93 ± 17.60 a |
|  | GH5 | 94.81 ± 3.68 a | 81.15 ± 5.23 b | 85.06 ± 0.27 b |
|  | GH6 | 39.31 ± 4.41 b | 21.63 ± 0.13 c | 58.49 ± 2.58 a |
|  | GH8 | 27.78 ± 2.18 b | 23.04 ± 1.46 b | 57.99 ± 10.17 a |
|  | GH9 | 104.86 ± 9.22 b | 107.08 ± 7.86 b | 177.66 ± 19.49 a |
|  | GH12 | 34.83 ± 2.96 b | 17.72 ± 1.83 c | 68.32 ± 13.62 a |
|  | GH45 | 0.00 ± 0.00 a | 0.09 ± 0.15 a | 1.15 ± 0.85 a |
|  | GH48 | 10.00 ± 2.49 ab | 5.33 ± 1.49 b | 12.93 ± 3.85 a |
|  | GH116 | 81.33 ± 5.44 ab | 68.08 ± 0.73 b | 90.85 ± 10.20 a |
|  | AA10 | 0.62 ± 0.54 a | 0.00 ± 0.00 a | 0.00 ± 0.00 a |
| plant-derived hemicellulose | GH2 | 512.85 ± 12.14 ab | 426.01 ± 8.75 b | 587.84 ± 81.13 a |
|  | GH10 | 93.42 ± 6.36 a | 71.19 ± 7.60 a | 112.54 ± 28.93 a |
|  | GH11 | 4.61 ± 2.19 a | 1.67 ± 1.90 a | 16.55 ± 13.71 a |
|  | GH26 | 66.81 ± 1.46 b | 59.18 ± 1.58 b | 111.65 ± 6.73 a |
|  | GH30 | 28.79 ± 6.12 a | 27.08 ± 2.02 a | 35.51 ± 3.72 a |
|  | GH36 | 157.35 ± 13.66 a | 142.25 ± 11.28 a | 169.86 ± 10.73 a |
|  | GH39 | 266.53 ± 16.02 a | 260.63 ± 2.21 a | 303.61 ± 58.83 a |
|  | GH43 | 16.36 ± 2.86 a | 10.19 ± 0.83 b | 16.91 ± 3.33 a |
|  | GH44 | 93.68 ± 4.86 a | 82.71 ± 5.17 a | 73.76 ± 19.81 a |
|  | GH51 | 216.30 ± 9.50 b | 238.54 ± 9.37 b | 344.66 ± 81.48 a |
|  | GH52 | 1.84 ± 0.57 a | 1.42 ± 1.07 a | 0.96 ± 0.74 a |
|  | GH54 | 29.23 ± 5.19 b | 16.28 ± 2.14 b | 59.25 ± 16.88 a |
|  | GH62 | 0.95 ± 0.27 a | 0.23 ± 0.39 a | 2.48 ± 1.65 a |
|  | GH67 | 51.96 ± 7.02 b | 42.89 ± 1.81 b | 87.92 ± 25.86 a |
|  | GH74 | 1834.98 ± 48.36 b | 2059.45 ± 27.12 a | 1838.54 ± 96.69 b |
|  | GH95 | 28.55 ± 3.93 b | 17.36 ± 1.63 b | 76.65 ± 35.10 a |
|  | GH115 | 25.63 ± 2.14 b | 21.91 ± 8.43 b | 52.77 ± 19.55 a |
|  | GH120 | 2.76 ± 0.65 a | 2.65 ± 0.98 a | 4.82 ± 1.25 a |
| plant-derived lignin | AA1 | 694.60 ± 15.34 a | 663.38 ± 26.11 a | 606.44 ± 34.37 b |
|  | AA2 | 187.14 ± 8.24 b | 187.33 ± 1.08 b | 232.19 ± 13.77 a |
|  | AA3 | 1386.21 ± 15.02 a | 1378.52 ± 22.50 a | 1407.79 ± 15.92 a |
|  | AA4 | 433.65 ± 4.73 a | 436.10 ± 8.60 a | 434.33 ± 6.49 a |
|  | AA5 | 620.57 ± 21.63 a | 606.93 ± 2.69 a | 620.10 ± 39.07 a |
|  | AA6 | 202.98 ± 1.83 b | 202.96 ± 10.64 b | 248.33 ± 10.05 a |
| fungi-derived chitin | GH16 | 153.27 ± 8.91 a | 131.19 ± 13.64 a | 152.24 ± 12.05 a |
|  | GH18 | 210.97 ± 5.42 ab | 184.41 ± 16.26 b | 235.40 ± 20.41 a |
|  | GH19 | 14.63 ± 1.37 b | 22.93 ± 4.59 a | 16.55 ± 0.78 b |
|  | GH20 | 149.28 ± 7.53 b | 159.13 ± 6.52 b | 202.81 ± 24.37 a |
|  | GH72 | 2.36 ± 0.27 a | 1.48 ± 0.43 b | 0.66 ± 0.02 c |
| fungi-derived glucans | GH17 | 63.17 ± 5.88 a | 74.81 ± 2.65 a | 68.97 ± 9.50 a |
|  | GH55 | 212.62 ± 15.24 a | 156.69 ± 3.71 b | 200.84 ± 17.45 a |
|  | GH64 | 15.22 ± 2.94a | 11.97 ± 4.89a | 11.76 ± 5.09a |
|  | GH81 | 2.18 ± 0.66a | 3.10 ± 0.25a | 2.00 ± 2.05a |
|  | GH128 | 19.72 ± 2.20 a | 10.46 ± 1.70 b | 21.79 ± 1.60 a |
| bacteria-derived peptidoglycan | GH23 | 831.94 ± 18.39 a | 835.88 ± 15.18 a | 852.89 ± 28.27 a |
|  | GH24 | 13.57 ± 3.17 a | 6.44 ± 0.72 b | 11.73 ± 2.91 a |
|  | GH25 | 88.70 ± 7.57 c | 114.83 ± 13.75 b | 162.69 ± 6.60 a |
|  | GH73 | 53.12 ± 5.83 b | 47.63 ± 2.79 b | 68.73 ± 4.82 a |
|  | GH102 | 60.99 ± 2.43 b | 82.27 ± 3.92 a | 81.42 ± 4.55 a |
|  | GH103 | 332.43 ± 16.15 b | 390.55 ± 19.52 a | 327.27 ± 14.28 b |
|  | GH104 | 1.77 ± 1.04 a | 1.84 ± 1.07 a | 1.60 ± 0.77 a |
|  | GH108 | 12.76 ± 1.69 a | 14.30 ± 0.49 a | 14.29 ± 2.76 a |
